# Simulation data for the analysis of Bayesian posterior significance and effect size indices for the two-sample t-test to support reproducible medical research

**DOI:** 10.1186/s13104-020-05291-z

**Published:** 2020-09-22

**Authors:** Riko Kelter

**Affiliations:** Department of Mathematics, University of Siegen, Siegen, Germany

**Keywords:** Bayesian significance and effect measures, Bayesian hypothesis testing, Student’s two-sample t-test, Welch’s two-sample t-test, Bayesian Biostatistics

## Abstract

**Objectives:**

The data presented herein represents the simulated datasets of a recently conducted larger study which investigated the behaviour of Bayesian indices of significance and effect size as alternatives to traditional p-values. The study considered the setting of Student’s and Welch’s two-sample t-test often used in medical research. It investigated the influence of the sample size, noise, the selected prior hyperparameters and the sensitivity to type I errors. The posterior indices used included the Bayes factor, the region of practical equivalence, the probability of direction, the MAP-based p-value and the e-value in the Full Bayesian Significance Test. The simulation study was conducted in the statistical programming language R.

**Data description:**

The R script files for simulation of the datasets used in the study are presented in this article. These script files can both simulate the raw datasets and run the analyses. As researchers may be faced with different effect sizes, noise levels or priors in their domain than the ones studied in the original paper, the scripts extend the original results by allowing to recreate all analyses of interest in different contexts. Therefore, they should be relevant to other researchers.

## Objective

The problems of p-values have been discussed in countless papers [1, 2], but for clinical research, only a few attractive alternatives have been proposed [3, 4]. Bayesian methods are one of the preferred solutions [5, 6]. This paper presents replication scripts which extend the results of a simulation study which investigated the behaviour of Bayesian posterior indices for the two-sample t-test. Indices were compared for their sensitivity to prior elicitation, sample size, error rates and noise.

**Table 1:** Overview of data files/data sets

Label	Name of data file/data set	File types (file extension)	Data repository and identifier (DOI or accession number)
Data file 1	Simulation script for medium Cauchy prior	R script file (.R)	Open Science Foundation [10.17605/OSF.IO/FBZ4S] [16]
Data file 2	Simulation script for wide Cauchy prior	R script file (.R)	Open Science Foundation [10.17605/OSF.IO/FBZ4S] [16]
Data file 3	Simulation script for ultrawide Cauchy prior	R script file (.R)	Open Science Foundation [10.17605/OSF.IO/FBZ4S] [16]
Data file 4	Simulation script for the influence of noise	R script file (.R)	Open Science Foundation [10.17605/OSF.IO/FBZ4S] [16]
Data file 5	Sensitivity analysis for type I error rates (using a wide Cauchy prior)	R script file (.R)	Open Science Foundation [10.17605/OSF.IO/FBZ4S] [16]

Results showed that indices differed substantially in their ability to control type I error rates and to detect an existing effect. Based on these results, the Bayes factor [7] and the ROPE [5] were isolated as suitable for the two-sample t-test.

The simulation data is generated by the provided replication scripts in the statistical programming language R [8] and should be relevant to other researchers. The original paper considered only four fixed effect sizes, and via the provided scripts the results of the original paper can be extended to different context. For example, if researchers expect effect sizes from $\delta = 0.4$ to $\delta = 0.6$, they can obtain the error rates, influence of noise and robustness to prior selection for different posterior indices and sample sizes for these specific effect sizes based on the provided scripts. This allows to select an index based on objective criteria. The scripts can be adapted easily to study the behaviour of posterior indices for other distributional settings (e.g. exponential or survival data). An example of the former reuse in the context of eating disorders is given in [9], where it is argued in favour of simulation-based insights into the behaviour of Bayesian posterior indices. An example of the latter reuse is given in [10], where the behaviour under preliminary testing for normality in the context of clinical trials is studied.

## Data description

In the study, pairs of datasets were simulated, consisting of two samples from the normal distribution. Four parameter settings were selected to model the presence of a small, medium, large and no effect size. For each of the four effect size settings, 10,000 datasets were generated. The simulations were repeated for different sample sizes n = 10 to n = 100 in steps of size 10 to investigate the influence of sample size on the indices. In each case, the traditional p-value, the Bayes factor [7], the 95% and full ROPE [4], the probability of direction [11], the MAP-based p-value [12] and the e-value [13] were computed. The Bayes factor was computed as the Jeffreys-Zellner-Siow Bayes factor for the null hypothesis $H_{0} :\delta = 0$ of no effect against the alternative hypothesis $H_{0} :\delta \ne 0$, see [14] for details. The calculated quantities thus were the Bayes factor and the posterior distribution, which was then used to compute the other indices.

The entire procedure was repeated in three different prior settings for the effect size $\delta$: The noninformative Jeffrey’s prior was always put on the standard deviation of the normal population, and a Cauchy prior $C\left( {0,\gamma } \right)$ was placed on the standardised effect size. The settings $C\left( {0,\sqrt 2 /2} \right)$, $C\left( {0,1} \right)$ and $C\left( {0,\sqrt 2 } \right)$ were selected, which correspond to a medium, wide and ultrawide prior on the effect size $\delta$. Data file 1 contains the R code to simulate the data for the setting of the medium prior, and data file 2 and data file 3 contain the code for simulation of the data for the wide and ultrawide prior settings. Note that instead of the raw simulation data, the replication scripts are provided here. These provide both the raw simulation data (the seed of the random number generator is fixed in each case to guarantee reproducibility) and the analysis results so that other researchers should benefit more from the replication scripts than from the raw simulation data.

The above procedure was repeated for the fixed sample size $n = 30$ to investigate the influence of noise, too. Gaussian noise $N\left( {0,\varepsilon } \right)$ was added to the group data $x$ and $y$, where $\varepsilon$ ranged from $\varepsilon = 0.5$ to $\varepsilon = 5$ in steps of $0.5$. Data file 4 includes the R script to simulate the data for the influence of noise.

The percentage of significant results was computed for samples of increasing size $n$ as the number of significant results divided by 10,000, which is an estimate for the type I error probabilities of the indices. The following significance thresholds were used in the files: A Bayes factor needed to be equal to or larger than three. The MAP-based p-value and traditional p-value were significant when $p_{MAP} < .05$ and p < 0.05. Details on the thresholds for the significance of the ROPE, probability of direction and e-value can be found in the original study [15]. Data file 5 contains the code to simulate the data for the type I error rates of each index. In each case, the wide Cauchy prior $C\left( {0,1} \right)$ was used to guarantee comparability of the results.

## Limitations

The scope of the study was limited to the setting of the two-sample Student’s and Welch’s t-test. Also, the results did not analyse the type II error rates, which could be essential in some contexts. Future studies should also investigate the behaviour of the indices for other methods which are used in medical research. These include regression models, survival analysis, and the analysis of variance. Additionally, the resulting type II error rates should be investigated by future work.

## Data Availability

The data described in this Data note can be freely and openly accessed on Open Science Foundation under 10.17605/OSF.IO/FBZ4S. Please see Table 1 and Ref. [16] for details and links to the data.

## Abbreviations

NHST: Null hypothesis significance testing
BF: Bayes factor
ROPE: Region of practical equivalence
PD: Probability of direction
MAP-based p-value: Maximum a posteriori based p-value
